# In Vivo Protein–Protein Binding Competition Assay Based on Split-GFP Reassembly: Proof of Concept

**DOI:** 10.3390/biom13020354

**Published:** 2023-02-11

**Authors:** Christophe Bignon, Sonia Longhi

**Affiliations:** Laboratory Architecture et Fonction des Macromolécules Biologiques (AFMB), UMR 7257, Centre National de la Recherche Scientifique (CNRS), Aix Marseille University, CEDEX 9, 13288 Marseille, France

**Keywords:** protein complementation assays, protein–protein interactions, intrinsically disordered proteins, fluorescence, binding competition assays

## Abstract

The split-green fluorescent protein (GFP) reassembly assay is a well-established approach to study protein–protein interactions (PPIs). In this assay, when two interacting proteins X and Y, respectively fused to residues 1–157 and to residues 158–237 of GFP, are co-expressed in *E. coli*, the two GFP halves are brought to sufficient proximity to reassociate and fold to recreate the functional GFP. At constant protein expression level, the intensity of fluorescence produced by the bacteria is proportional to the binding affinity of X to Y. We hypothesized that adding a third partner (Z) endowed with an affinity for either X or Y would lead to an in vivo competition assay. We report here the different steps of the set-up of this competition assay, and define the experimental conditions required to obtained reliable results. Results show that this competition assay is a potentially interesting tool for screening libraries of binding inhibitors, Z being either a protein or a chemical reagent.

## 1. Introduction

**Figure 1:**
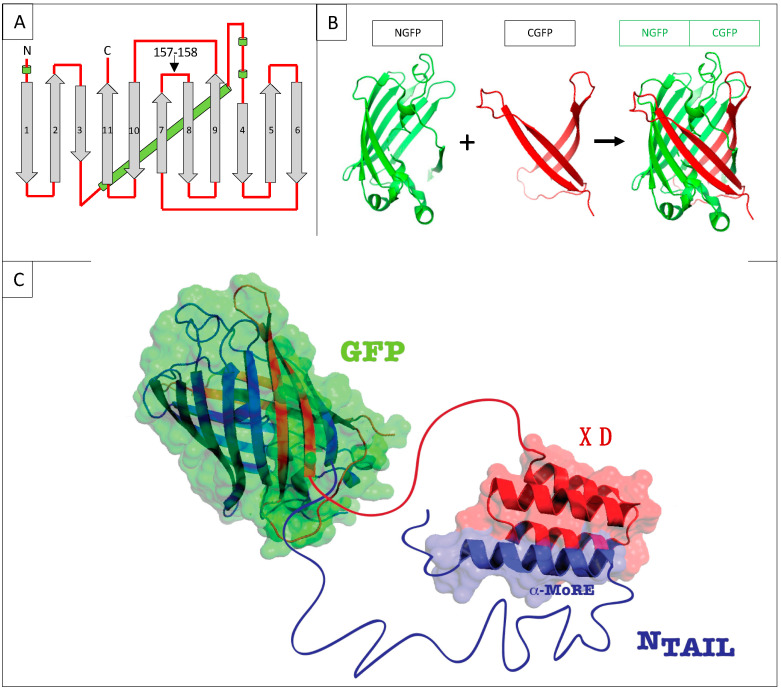
(**A**) GFP topology diagram. β-strands are numbered and represented as grey arrows, α-helices are represented as green cylinders and loops as red lines. The internal α-helix containing the chromophore is represented behind the structure. N, N-terminus. C, C-terminus. The cutting point between residues 157 and 158 is indicated. (**B**) Split-GFP reassociation. Cartoon representation illustrating how NGFP (1–157) and CGFP (158–238) reassociate to recreate the fluorescent protein. (**C**) Cartoon representation of the complex resulting from the assembly of NGFP-X and Y-CGFP fusion proteins, in this example X being measles virus N_TAIL_, and Y XD.

Protein–protein interactions (PPIs) are central in physiological as well as pathological cellular events. Therefore, many in vitro and in vivo techniques for the detection of PPIs have been devised (for reviews see [1,2]). In turn, these PPI methods have provided an asset for setting up competition assays designed for use in a large panel of applications including screening of potential protein or chemical inhibitors of a given interaction, evaluating the affinity of the interaction, or testing variants of one of the two interacting proteins. This has been the case for NMR [3], FRET [4], yeast two-hybrid [5], and split-luciferase [6], just to name a few. However, to the best of our knowledge, no competition assay has been set-up so far on the basis of the bipartite split-GFP reassembly assay devised in Lynne Regan’s lab in the early 2000 [7,8]. GFP is a β barrel made of 11 β strands. The technique is based on splitting GFP into two parts by cutting between residues 157 and 158 located within a loop (Figure 1A). This cutting generates an N-terminal half made of β strands 1 to 7 (NGFP, residues 1–157) and a C-terminal half made of β strands 8 to 11 (CGFP, residues 158–237) (Figure 1B). Two proteins X and Y are then respectively fused to the C-terminus of NGFP and to the N-terminus of CGFP, and the two fusion proteins are co-expressed in *E. coli*. If X and Y bind to each other, the two GFP halves are brought to sufficient proximity to reassociate and fold to recreate the functional GFP (Figure 1B,C). We have previously shown that the amount of fluorescence generated by the reconstituted GFP depends on several factors such as the GFP variant used, X to Y binding affinity, solubility and expression level of NGFP-X and Y-CGFP fusions, and expression temperature [9]. Assuming a constant soluble expression of the two fusion proteins, the intensity of the fluorescence emitted by the bacteria is proportional to the interaction strength between X and Y, which provides an easy way to quantify affinity differences between protein pairs.

**Figure 2:**
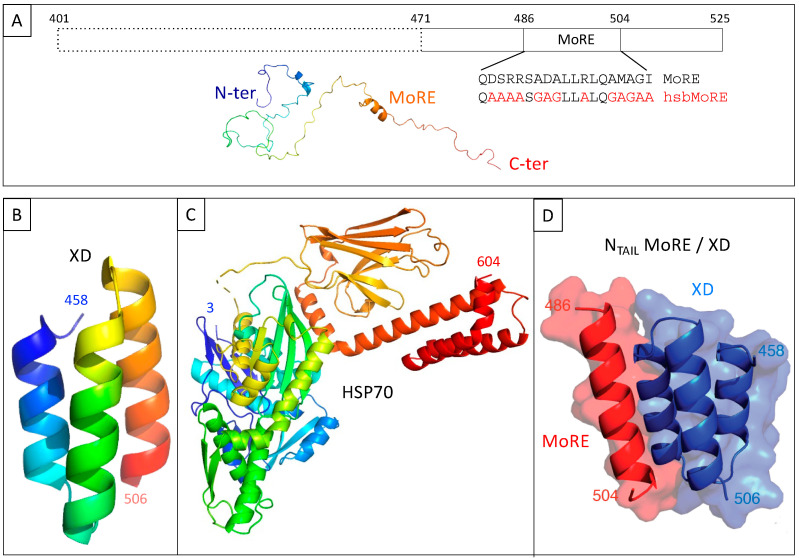
(**A**) Schematic representation of measles virus N_TAIL_ (**upper** panel) and cartoon representation of an N_TAIL_ conformer generated using Flexible-Mecano [22]. (**B**) Ribbon representation of the crystal structure of measles virus XD (PDB code 1OKS). (**C**) Cartoon representation of the crystal structure of HSP70 based on pdb codes 1HJO and 4JNF. The relative orientation of the two hsp70 domains (i.e., amino acids 3–382 and amino acids 389–610) is based on the structure of a form encompassing residues 1–554 (pdb code 1YUW). (**D**) Structure of the complex between measles virus XD (blue) and of the MoRE of N_TAIL_ (red, aa 486–504) (PDB code 1T6O). The buried surface area of the XD/MoRE complex is 634 Å^2^.

We have used this technique over the past ten years to investigate the interaction between the intrinsically disordered C-terminal domain of the measles virus nucleoprotein (N_TAIL,_ aa 401–525 of N protein) (Figure 2A) [10] and two of its binding partners, the C-terminal X domain of the viral phosphoprotein (XD, aa 458–507 of P protein) (Figure 2B) [11,12] and the major inducible 70 kDa heat shock protein (HSP70) (Figure 2C) [13,14,15]. XD is a small (~6 kDa) triple α-helical bundle (Figure 2B) whose binding to N_TAIL_ triggers α-helical folding within a short molecular recognition element (MoRE) of N_TAIL_ (aa 486–504) (Figure 2D) [11,12] while the rest of N_TAIL_ remains disordered in the complex [16]. Contrary to the N_TAIL_/XD complex, no structural information is available on the N_TAIL_/HSP70 complex.

In the past decade, we have used the split GFP complementation assay in random mutagenesis experiments aimed at deciphering the molecular determinants of the interaction between N_TAIL_ and XD [17], to demonstrate the self-inhibitory effect of the N-terminal “fuzzy” (i.e., disordered) appendage of N_TAIL_ on its binding to XD [18], to analyze the impact of varying the α-helicity of the N_TAIL_ molecular recognition element (MoRE) on binding to XD and HSP70 [19,20], to investigate the molecular mechanisms underlying N_TAIL_ binding to XD and to HSP70 [19,20], to create an artificial peptide with an increased affinity for HSP70 [19,20], and to experimentally test a software designed for generating artificial intrinsically disordered proteins [21]. From those previous studies, we inferred that in contrast with the complex with XD, the MoRE of N_TAIL_ does not adopt an α-helical conformation when bound to HSP70 [19,20].

In a recent paper summarizing our long-standing practice of the technique over years, we have analyzed different parameters that were shown to be critical for obtaining reliable and quantitative results [9]. Starting from that study, we reasoned that adding a third protein Z to the bipartite reaction (NGFP-X Y-CGFP reassembly) could lead to the set-up of a binding competition assay provided that the third protein is able to interact with one of the two proteins of the split-GFP complex. Note that we use the term competition rather than displacement because the reassociation of GFP is irreversible [8].

Here, we describe the setting up of this in vivo PPI competition assay, and discuss the potential traps we came across. To that end, we have used the above-described N_TAIL_/XD and N_TAIL_/HSP70 model interactions. For the N_TAIL_/XD interaction, we previously showed that a shorter form (i.e., 55 residue long) of N_TAIL_ (hereafter referred to as 471) encompassing the MoRE and devoid of the fuzzy N-terminal appendage, provided more fluorescence than full-length N_TAIL_ (125 residues), although the K_D_ of the binding reaction was not determined [18]. Therefore, we have used 471 rather than full-length N_TAIL_ in the present study so as to potentially better detect fluorescence decreases that were expected to occur in case of binding competition (Figure 2A). Similarly, we also demonstrated that an artificial peptide called hsbMoRE (HSP70 super binder MoRE) and derived from wild-type MoRE (Figure 2A), binds HSP70 with an apparent affinity higher than that of its wild-type counterpart and of 471, and consequently elicits more fluorescence than 471 when used with HSP70 in a bipartite split-GFP reassembly assay [19]. Therefore, in the present study, we have taken advantage of the affinity difference between 471 and hsbMoRE for HSP70 (hereafter simply referred to as HSP) to compare the effect of adding a competitor to a low (471/HSP) and high (hsbMoRE/HSP) affinity interaction involving a common protein (HSP). In practice, three interacting pairs were tested for competition, namely, 471/XD, 471/HSP, and hsbMoRE/HSP. Results advocate for the validity of the approach and provide a proof of concept.

## 2. Materials and Methods

### 2.1. Expression Constructs

The constructs encoding the half GFP fusion proteins have already been described [17]. All NGFP fusions are named after their fusion partner: NGFP-471 (N4), NGFP-hsbMoRE (NM), NGFP-HSP (NH). NGFP fusions are all N-terminally His-tagged. Some CGFP fusions are not His-tagged, with this being specified in the text whenever necessary. In the negative control are co-expressed (i) CGFP fused to one of the proteins of interest, (ii) NGFP alone, as encoded by a construct in which the gene fragment encoding NGP is fused to the Ntail coding sequence with two in-frame stop codons between the two (NGFP-stop, NS), (iii) a competitor (see below) or the empty p17Tet vector (TS) (see below).

The vector for the expression of the competitor, referred to as p17Tet, was generated as follows. Genomic DNA was extracted from *E. coli* Origami cells, which are spontaneously resistant to tetracycline, and used as template with primers T5Hind3 (ACTACCGCATTAAAGCTTCTCGACATCTTGGTTACCGT) and T3AhdI (TCCATAGTTGCCTGACTCCCCGTCCGCGGAATAACATCATTTGG) for PCR amplifying the tetracycline operon (tetA, tetR). After DpnI treatment, the PCR product was digested with HindIII and AhdI (NEB) and then ligated to pDEST17O/I [23] that had been digested with the same enzymes and gel-purified to separate the vector backbone from the ampicillin resistance gene. Using AhdI as 3′ cloning site keeps the last, non-coding, 65 bp of the ampicillin resistance gene in the final construct. The sequence and main features of p17Tet are shown in Supplementary Text S1.

The negative control of the competitor (p17Tet-stop, TS) was constructed as NS (see above) by inserting the sequence coding for N_TAIL_ with two in-frame stop codons (TAATAA) at the beginning of the sequence just after the 5′ Gateway attb1 cloning site.

The constructs expressing the different competitors used in this study, namely 471 (T4), XD (TX), HSP (TH), hsbMoRE (TM), and PNT3 [24] (TP), were obtained by PCR amplifying the respective coding sequence of the competitors using primers flanked by the attb1 and attb2 sequences, and then inserting the PCR products in p17Tet by BP and LR reaction. The resulting constructs drive the expression of N-terminally hexahistidine tagged proteins.

### 2.2. Split-GFP Reassembly Competition Assay

T7 *E. coli* cells were co-transformed with the two plasmids of the split-GFP reassembly assay using either sg100 or fr GFP variants [9] and with either p17Tetstop or p17Tet expressing a competitor, plated on LB-agar plates containing 100 µg/mL ampicillin, 50 µg/mL kanamycin and 15 µg/mL tetracycline, and allowed to grow overnight at 37 °C. The next day, a few colonies were scraped off and used to seed 4 mL of LB containing the same antibiotics in 24-wells deep-well plates. After one night at 37 °C under shaking at 200 rpm, 100 µL of pre-culture were used to seed 4 mL of TB containing the same three antibiotics in 24-wells deep-well plates. The culture was incubated for one and a half hour at 37 °C under shaking at 200 rpm. IPTG (0.5 mM) and arabinose (2%) were added, and the deep-well plate was incubated for different periods ranging from overnight to 72 h at 17 °C. The deep-well plate was then span for 2 min at 2000 g and the culture medium was discarded. One milliliter of PBS was added to each well and the cell pellet was resuspended. The optical density at 600 nm and the fluorescence of 100 µL of a 1/10 dilution of this suspension were measured using a TECAN GENios Plus plate reader (TECAN France, Tour Swiss Life, 1 Bd Marius Vivier Merle, 69003 Lyon, France) and clear bottom, black 96-well plates. Data were processed using Excel. The fluorescence (in arbitrary units) was divided by the OD_600_ and the mean and standard deviation of the ratio of each triplicate was calculated. Expression of the different fusion proteins were analyzed by SDS-PAGE as described in [9].

## 3. Results

### 3.1. Generation of the Construct for the Expression of the Competitor and Principle of the Competition Assay

**Figure 3:**
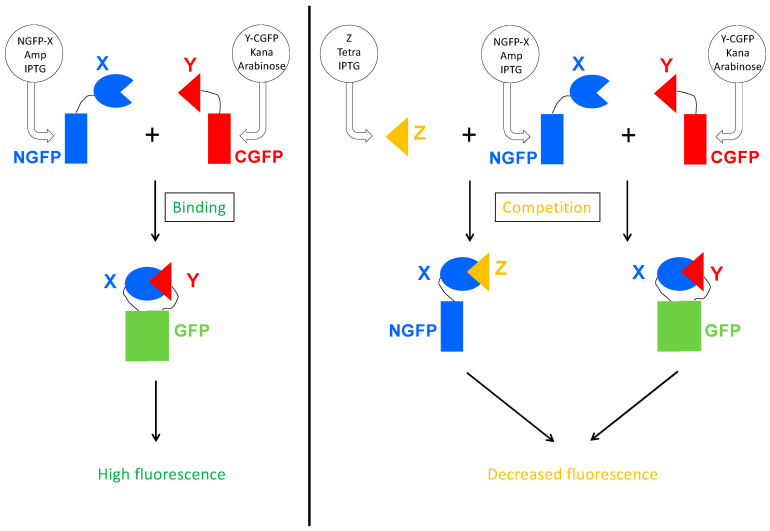
Principle of the Split-GFP-based competition assay. On the **left** panel is represented the split-GFP reassembly assay. Two proteins X and Y known to interact are respectively fused to the N-terminal (NGFP) and to the C-terminal part of GFP (CGFP), and co-expressed in *E. coli* by their respective plasmid. The plasmid coding for NGFP fusion confers ampicillin resistance and expression is induced by IPTG. The plasmid coding for CGFP fusion confers kanamycin resistance and expression is induced by arabinose. When protein X binds to protein Y, their two GFP halves are brought together and reconstitute the functional GFP. On the **right** panel, a competitor Z is co-expressed with NGFP-X and Y-CGFP. The plasmid coding for Z confers tetracycline resistance and Z expression is induced by IPTG. Z binds to X, which reduces the amount of NGFP-X fusion protein available for binding to Y-CGFP, and hence reduces the amount of fluorescence produced by the bacteria.

As a first step, we built a plasmid for expressing the competitor protein. The plasmid had to fulfill a few criteria. Expression of the competitor should be inducible and the plasmid should be selectable during cell transformation, and then maintained throughout the whole experiment. Since finding an inducible plasmid with an origin of replication compatible with those of the two plasmids expressing the GFP halves proved to be difficult, we eventually swapped the ampicillin resistance gene of the Gateway vector pDEST17O/I [23] we routinely use, with the tetracycline resistance operon since the two plasmids of the split-GFP assay already provide ampicillin and kanamycin resistance, respectively. The new vector, referred to as p17Tet (Supplementary Text S1), drives the inducible expression of the competitor. Binding of the competitor to the NGFP-X fusion protein is expected to reduce the amount of NGFP-X available for binding to Y-CGFP thus leading to a reduced fluorescence (Figure 3).

Note that in the present study, we used the original GFP reporter sg100 because we showed in a previous paper that it provided the best results [9].

### 3.2. Competing the 471/XD Interaction Using 471 as Competitor

**Figure 4:**
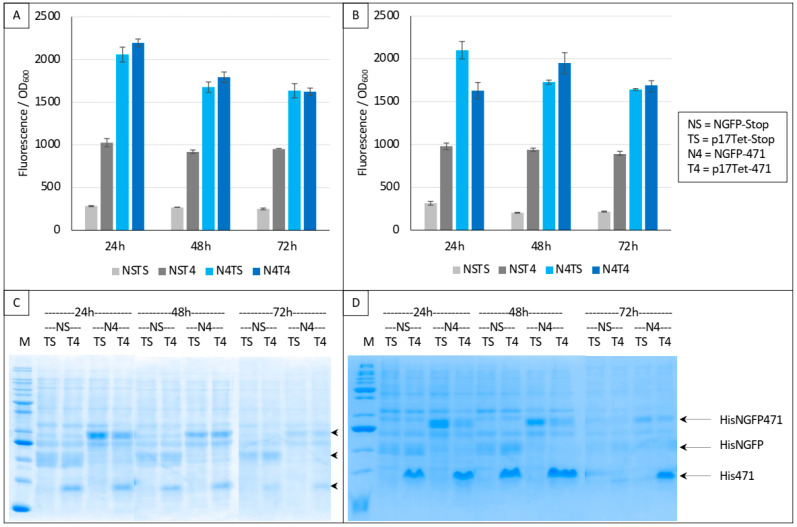
Split-GFP reassembly competition assay using NGFP-471, XD-CGFP, and His-471 as competitor. (**A**,**B**) Fluorescence data obtained in two independent experiments, each performed in triplicate for 24, 48, or 72 h. NSTS and NST4 are the two negative controls. See main text for a description of NSTS, NST4, N4TS, and N4T4. (**C**,**D**) SDS-PAGE analysis of (**A**,**B**), respectively. M, molecular mass markers (200, 150, 100, 85, 60, 50, 40, 30, 25, 20, 15, 10 kDa).

In a first trial, we ran experiments for up to 72 h based on the interaction between 471 and XD. Two negative controls were used in these experiments: NSTS, in which the N-terminal part of GFP (NGFP, “NS”) and the C-terminal part of GFP fused to XD (XD-CGFP) are co-expressed along with p17Tet expressing no competitor owing to the presence of two in-frame stop codons at the beginning of the Ntail coding sequence (p17Tet-stop, “TS”) (see Section 2), and NST4, in which NGFP, XD-CGFP, and the competitor 471 (expressed by p17Tet-471, “T4”) are co-expressed. The positive control (N4TS) was obtained by co-expressing NGFP fused to 471 (NGFP-471, “N4”) and XD-CFGP in the presence of the p17Tet vector that does not encode the competitor (p17Tet-stop, “TS”). Finally, the competition was assessed by co-expressing NGFP-471, XD-CFGP, and 471 (“N4T4”). Results of two independent experiments are reported in Figure 4. Two types of results could be distinguished which, in no case, met expectations. The first type of results, obtained more often, did not reflect any competition effect (Figure 4A,B, 48 h, 72 h). The second type of results was characterized by some competition effect (Figure 4B, 24 h). Surprisingly, the most reproducible results were provided by the two negative controls NSTS and NST4 that, unexpectedly, exhibited two different fluorescence values, NST4 being systematically higher than NSTS. Note that the absence of competition (type 1 results) could not be ascribed to a lack of competitor, since the latter was visible in all cases in SDS-PAGE (T4 lanes in Figure 4C,D). An explanation for the difference in fluorescence values of the two negative controls and for the absence of competition can be inferred from our previous observations that XD-CGFP undergoes degradation when expressed alone [9], and is detailed below.

**Figure 5:**
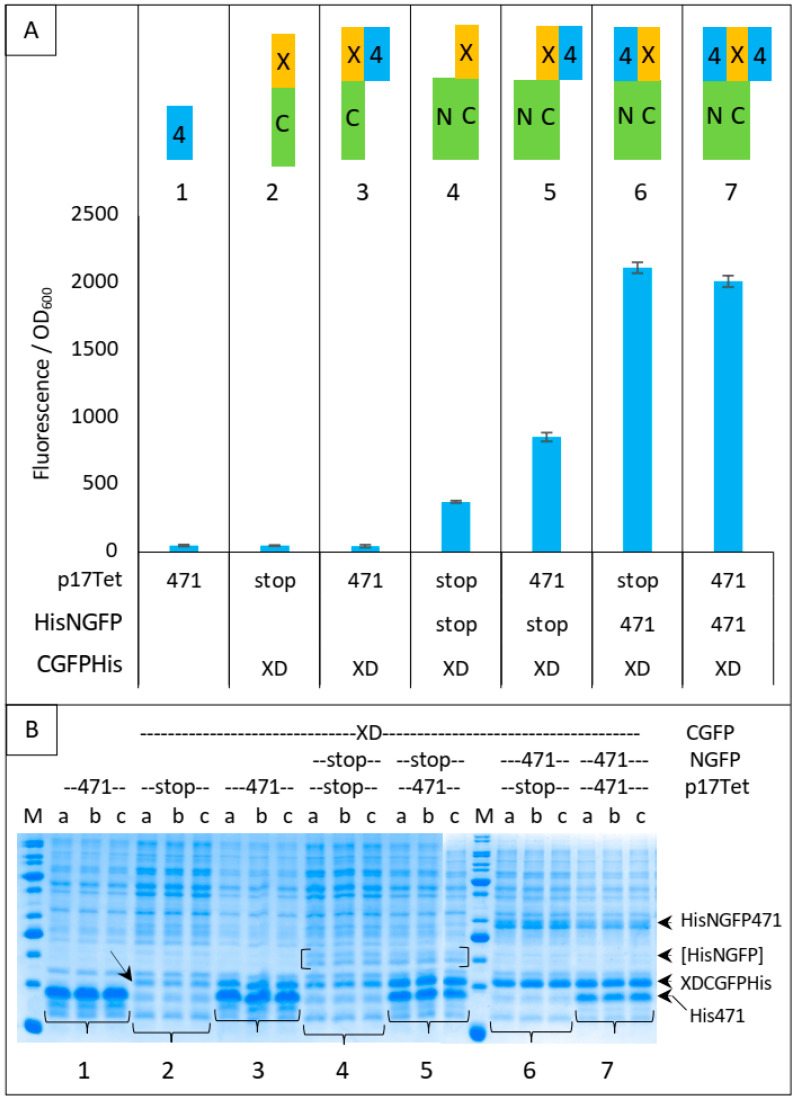
XDCGFP bound to 471 and/or NGFP is protected from degradation as assessed by fluorescence measurements after one night at 17 °C and SDS-PAGE analysis. In this experiment, XDCGFP is his-tagged. (**A**) **Top** panel: the different proteins and protein combinations, numbered from 1 to 7, are illustrated by colored rectangles: 471 = 4 in blue rectangle, XD = X in yellow rectangle, CGFP = C in green rectangle, NGFP = N in green rectangle. Fusion proteins XC and 4N are represented as fused rectangles. To avoid ambiguity, competitor 471 bound to XD is arbitrarily represented on the right of XD, whereas NGFP471 bound to XDCGFP is arbitrarily represented on the left of XDCGFP. **Bottom** panel: fluorescence data obtained with conditions 1 to 7 are represented just below the corresponding protein or protein combinations 1 to 7. (**B**) SDS-PAGE analysis of proteins expressed in (**A**). Triplicates were loaded individually (a, b, c) to assess the reproducibility of each loading. The lowest steady-state level of XD-CGFP is indicated by an oblique arrow (condition 2). His-NGFP is indicated by square brackets. Numbers 1 to 7 below the gels refer to the different combinations of (**A**). The different fusion proteins are indicated on the right of the gels. M, molecular mass markers (200, 150, 100, 85, 60, 50, 40, 30, 25, 20, 15, 10 kDa).

In Figure 5, we have analyzed in more detail the protective effect of different combinations of proteins against XD-CGFP-His degradation by measuring the fluorescence emitted by different protein complexes after 24 h of protein expression (Figure 5A) and by assessing the intensity of the XD-CGFP-His band on gel (Figure 5B). Fluorescence values are informative only when the two GFP halves are co-expressed (Figure 5, conditions 4 to 7). As shown in Figure 5A, the lowest fluorescence value was obtained when XD-CGFP-His was co-expressed with NGFP (condition 4).

The mere addition of 471 to condition 4 provided some degree of protection to XD-CGFP-His against degradation (compare the fluorescence provided by conditions 4 and 5 in Figure 5A), but the protection provided by the fusion protein NGFP-471 (condition 6) was higher than that provided by the separate co-expression of its constitutive parts (i.e., 471 + NGFP, condition 5). As expected, and although SDS-PAGE is not as quantitatively reliable as fluorescence measurements, Figure 5B confirms a correlation between XD-CGFP-His band intensities and fluorescence values (Figure 5A) for conditions 4 to 7. In the absence of one of the two GFP halves (conditions 2 and 3), there is no fluorescence and hence only XD-CGFP-His band intensity on gel can be used to assess the protection effect. Here again, XD-CGFP-His was barely detectable when expressed alone (oblique arrow in Figure 5B, condition 2), and co-expression with 471 provided a certain degree of protection (Figure 5B, condition 3), a result confirming the observation made above by comparing fluorescence data, as well as gel data, of conditions 4 and 5.

**Figure 6:**
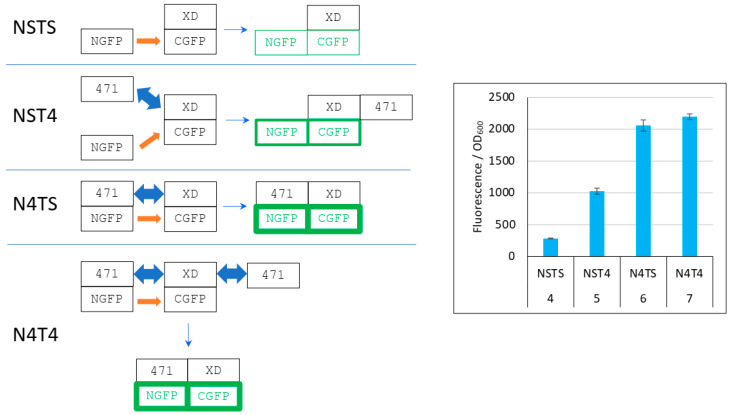
Explanation of Figure 4 results. **Left** panel, cartoon explaining the different protein interactions and the corresponding fluorescence results. XD-CGFP and NGFP-471 fusion proteins are represented as linked rectangles. Non-fluorescent proteins are shown in black. Fluorescent proteins are in green. The green border thickness is proportional to the intensity of fluorescence. The thin blue arrow points towards the final complex. Thick blue double arrow: fast, reversible interaction. Orange arrow: slow, irreversible interaction. To avoid any ambiguity, free 471 bound to XD is represented on the right of XD, whereas NGFP-471 bound to XD-CGFP is represented on the left of XD-CGFP. See main text for a description of NSTS, NST4, N4TS and N4T4. **Right** panel, Fluorescence data of a triplicate experiment run overnight. Numbers 4 to 7 of the *x*-axis refer to the experimental conditions described in Figure 5.

On the basis of the different levels of XD-CGFP protection against degradation by the different interacting partners described in Figure 5, we propose in Figure 6 an explanation of the results reported in Figure 4.

In the first negative control (NSTS), NGFP interacts slowly and irreversibly with CGFP, which leads to low fluorescence background because (*i*) of the slow association rate constant (k_on_) between NGFP and CGFP, (*ii*) unbound XD-CGFP undergoes degradation (condition 4 in Figure 5 and Figure 6). In the second negative control (NST4), 471 interacts quickly and reversibly with XD-CGFP. NGFP interacts slowly and irreversibly with this already preformed 471/XD-CGFP complex. By reconstituting the full complex, these two independent interactions protect XD-CGFP from degradation, which significantly increases the fluorescence provided by NST4 with respect to NSTS (condition 5 in Figure 5 and Figure 6). In the positive control N4TS, NGFP-471 interacts quickly (471/XD binding) and irreversibly (NGFP/CGFP binding) with XD-CGFP, which produces more fluorescence than NST4 background (condition 6 in Figure 5 and Figure 6) because the NGFP-471 fusion protein is more effective than its two constitutive parts NGFP and 471 expressed separately at generating folded and fluorescent GFP (Figure 5A). In the presence of competitor (N4T4, condition 7 in Figure 5 and Figure 6), not only NGFP-471 interacts with CGFP-XD, thus leading to high fluorescence values, but also 471 interacts quickly and reversibly with XD-CGFP, which protects XD-CGFP from degradation (as in condition 3 in Figure 5B) until 471 is displaced by NGFP-471 that binds XD-CGFP irreversibly. This second pathway further increases the already high fluorescence, which explains why N4T4 can yield higher fluorescence than N4TS, and so why no competition effect is observed (type 1 result). In practice, however, in independent experiments we observed both competing (type 2 result) and potentializing effects of 471 on the binding of NGFP-471 to XD-CGFP (type 1 result) (see Figure 4). We cannot explain why there is no clear trend except noting the two contradictory effects of 471 illustrated by N4T4: on the one hand, it can increase the fluorescence by protecting XD-CGFP from being degraded (as illustrated by NST4 providing more fluorescence than NSTS), but on the other hand it can also decrease the fluorescence by competing with NGFP-471 for binding to XD-CGFP. Why one or the other of these two opposite effects is prevalent in one experiment and not in another remains elusive, but type 2 results are explained by the same mechanism as that considered for type 1 results except that, in that case, it is the competition effect of 471 that is prevalent over its protective effect on XD-CGFP degradation.

### 3.3. Competing the 471/XD Interaction Using XD as Competitor

**Figure 7:**
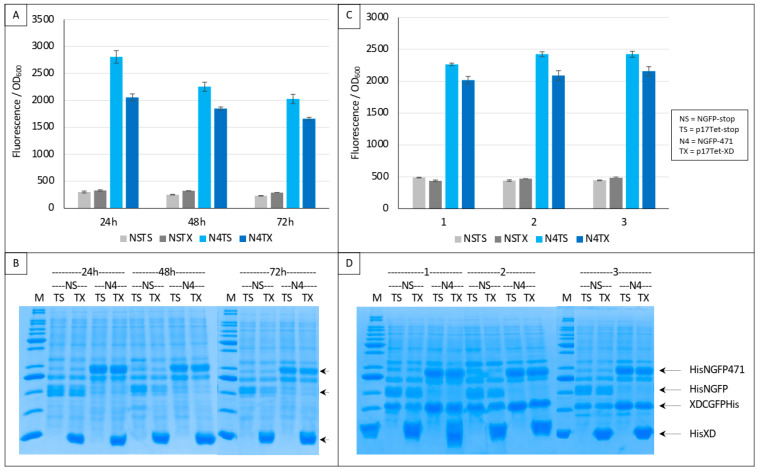
Split-GFP reassembly competition assay using NGFP-471, XD-CGFP, and His-XD as competitor. (**A**) Fluorescence data as obtained from an experiment performed in triplicate for 24, 48 or 72 h. NSTS and NSTX are the two negative controls. TX is free XD used as competitor. See main text for a description of NSTS, NSTX, N4TS and N4TX. (**B**) SDS-PAGE analysis of (**A**). M, molecular mass markers (200, 150, 100, 85, 60, 50, 40, 30, 25, 20, 15, 10 kDa). (**C**) Fluorescence data as obtained from three independent experiments (labeled 1, 2, 3 on the *x*-axis), each performed in triplicate for 24 h. The difference between (**A**,**C**) is that experiments shown in (**C**) used a His-tagged version of XD-CGFP. (**D**) SDS-PAGE analysis of (**C**). M, molecular mass markers (200, 150, 100, 85, 60, 50, 40, 30, 25, 20, 15, 10 kDa).

Irrespective of the underlying reasons for the two types of results described in Figure 4, Figure 5 and Figure 6, if the mechanism proposed in Figure 6 is correct, then completely different results should be obtained by using XD instead of 471 as competitor because NGFP-471 does not undergo degradation when expressed alone contrary to XD-CGFP [9]. These experiments used the same protein combinations as those used in experiments reported in Figure 4, Figure 5 and Figure 6 except that the competitor was XD (“TX”) instead of 471 (“T4”). Results, reported in Figure 7, confirmed our hypothesis.

The two negative controls NSTS and NSTX were now both low and identical, and addition of the competitor decreased the fluorescence as expected (Figure 7A). Gel analysis (Figure 7B) indicated a high expression of competitor XD compared to that of the two NGFP fusions, further explaining the competition observed. Comparable results were obtained when a His-tagged version of XD-CGFP was used (Figure 7C). The use of His-tagged XD-CGFP again underlined the proportionality between fluorescence values and XD-CGFP band intensity on gel (Figure 7D).

The different behavior of 471 (Figure 4, Figure 5 and Figure 6 and Supplementary Figures S1 and S2) and of XD (Figure 7 and Supplementary Figure S3) when used as competitors is explained in detail in Figure 8.

As it was the case in Figure 6, in the first negative control (NSTS), NGFP interacts slowly and irreversibly with CGFP, leading to low fluorescence background for the reasons explained above. In the second negative control NSTX, and in contrast with NST4 (Figure 6), XD-CGFP is not protected from degradation because XD, unlike 471, does not interact with XD-CGFP. Consequently, the fluorescence provided by NSTX is identical to that provided by NSTS whereas the fluorescence provided by NST4 was higher than that provided by NSTS. In positive control N4TS, NGFP-471 interacts quickly and irreversibly with XD-CGFP, which leads to high fluorescence signal above NSTS and NSTX backgrounds. In the presence of competitor XD, there are three cumulative reasons explaining why N4TX provides less fluorescence than N4TS. First, while NGFP-471 interacts with CGFP-XD to generate a high fluorescence signal, XD also interacts with NGFP-471 (and potentially faster than XD-CGFP because the latter is bigger and hence diffuses more slowly than XD). Thus, there is a competition between XD and XD-CGFP for binding to NGFP-471, which results in a decreased fluorescence compared to N4TS. Second, because of the competition performed by XD for binding to NGFP-471, more unbound XD-CGFP undergoes degradation than in the case of N4TS. Third, as in the case of NSTX, XD-CGFP is not protected from degradation because XD does not bind XD-CGFP.

### 3.4. Competing the Ntail/HSP Interaction Using HSP as Competitor

**Figure 8:**
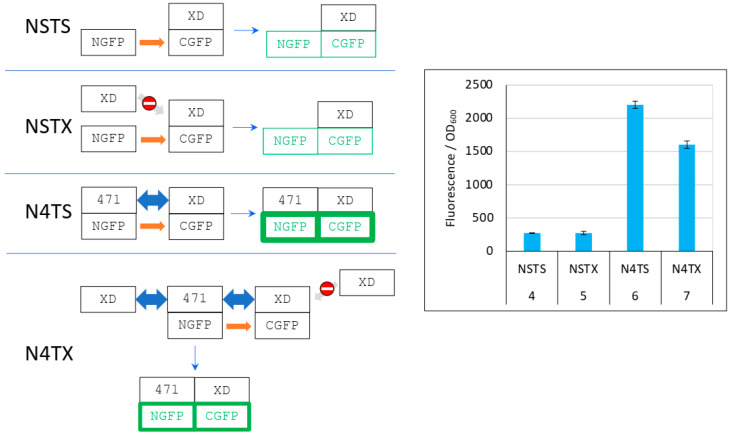
Explanation of Figure 7 results. **Left** panel, cartoon explaining the different protein interactions and the corresponding fluorescence results. See Figure 6 for details and main text for a description of NSTS, NSTX, N4TS, and N4TX. The light grey arrow with a no-way symbol indicates an absence of interaction. **Right** panel, fluorescence data of a triplicate experiment run overnight. Numbers 4 to 7 of the *x*-axis refer to the experimental conditions described in Figure 5.

**Figure 9:**
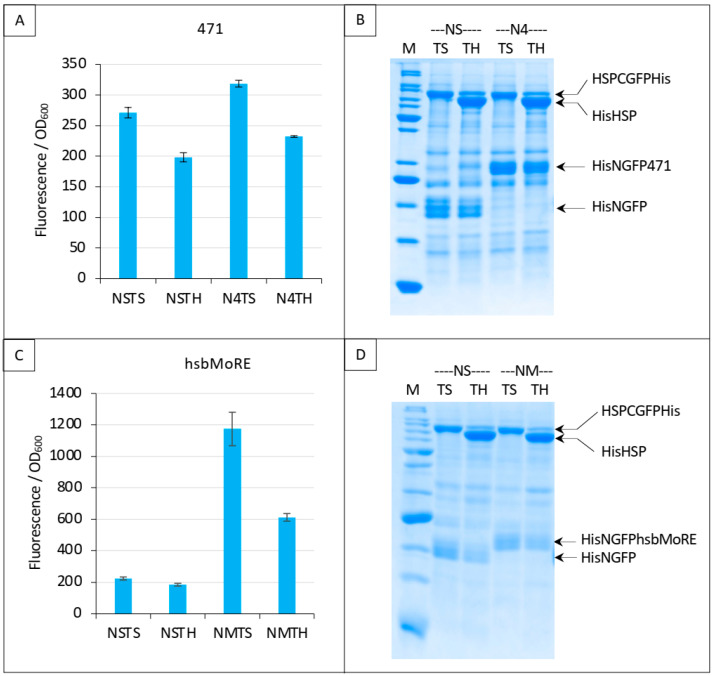
Split-GFP reassembly competition assay using NGFP-471 (**A**,**B**) or NGFP-hsbMoRE (**C**,**D**) with HSP-CGFP, and HSP as competitor. (**A**,**C**) Fluorescence data. (**B**,**D**) SDS-PAGE analysis of protein expression. See main text for a description of NSTS, NSTH, N4TS and N4TH. M, molecular mass markers (200, 150, 100, 85, 60, 50, 40, 30, 25, 20, 15, 10 kDa).

The experiments described above used XD as the interacting partner, whose K_D_ toward N_TAIL_ is ~3 μM [25]. We next tested the competition assay using a weaker interaction, namely the interaction with HSP, another interacting partner of N_TAIL_ with a K_D_ of ~70 μM [13,14,15]. As in the case of the interaction with XD, instead of full-length N_TAIL_ we first used the N-terminally truncated N_TAIL_ variant 471 that provided higher fluorescence values [18]. Note that the K_D_ of the interaction between N_TAIL_ variant 471 and HSPhas not been determined yet. The competitor we tested with this pair of proteins was HSP (“TH”, Figure 9). On the basis of the results reported in Figure 4, Figure 5, Figure 6, Figure 7 and Figure 8, experiments were run for 24 h only. As expected, much less fluorescence was detected with HSP than with XD (Figure 9A and Supplementary Figure S4A). In addition, high fluorescence values for negative controls NSTS and NSTH were obtained. As a result of this high background, even if a fluorescence decrease was observed in the presence of competitor (N4TH) and in spite of the fact that competitor HSP was indeed well expressed (Figure 9B), the competition effect could not be considered as specific.

**Figure 10:**
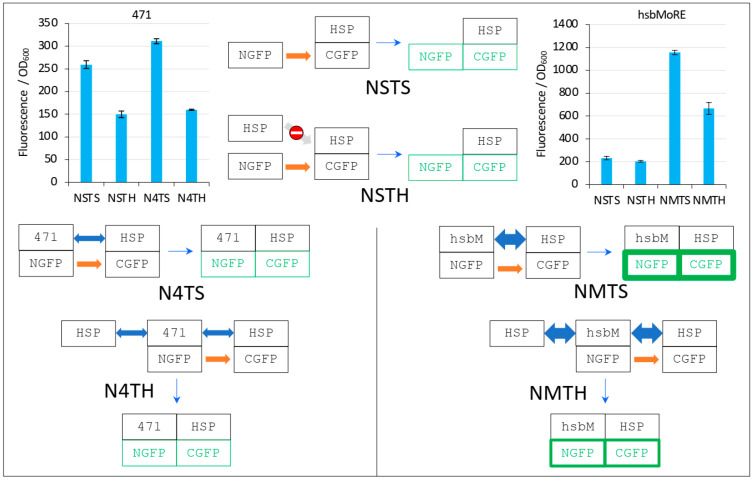
Explanation of Figure 9 results. The two top left and right bar plots are fluorescence data obtained by using 471 and hsbMoRE, respectively. See Figure 6 for details and main text for a description of NSTS, NSTH, N4TS, and N4TH. The light grey arrow with a no-way symbol indicates an absence of interaction.

In order to increase fluorescence values and signal to noise ratios, we replaced 471 with hsbMoRE, a peptide we derived from N_TAIL_ MoRE that is endowed with a higher affinity for HSP [19] although the K_D_ of the hsbMoRE/HSP binding reaction has not been determined yet. Results show that swapping 471 with hsbMoRE increased the fluorescence signal almost four times (from about 300 to about 1200) while, as expected, the fluorescence of negative controls NSTS and NSTH remained unchanged (Figure 9C). In light of the results obtained with hsbMoRE (Figure 9C), those obtained using 471 (Figure 9A) could now be reanalyzed retrospectively: the competition observed (i.e., the difference between N4TS and N4TH, Figure 9A) did exist but was masked by the high background of NSTS and NSTH, which calls into questions the reliability of negative controls in the case of low affinity interactions in split-GFP reassembly assay, as previously discussed [9]. Of note, hsbMoRE provided more fluorescence (NMTS, Figure 9C) than 471 (N4TS, Figure 9A) although its steady-state level in the cell was lower (comparing His-NGFP-471 bands in N4 lanes in Figure 7B and His-NGFP-hsbMoRE bands in NM lanes in Figure 9D), suggesting that affinity is more important than protein expression levels to account for fluorescence results. A detailed explanation of the results reported in Figure 9A,C is provided in Figure 10 and discussed below.

In the first negative control NSTS, NGFP slowly interacts with HSP-CGFP, which leads to low fluorescence background (a fluorescence to OD_600_ ratio around 200, Figure 10). This is surprising because we have shown that NSTS is low when XD-CGFP is used (a fluorescence to OD_600_ ratio also around 200 in Figure 4, Figure 5, Figure 6, Figure 7 and Figure 8) because XD-CGFP undergoes degradation. Since HSP-CGFP does not undergo degradation when expressed alone (Figure 9B,D and Supplementary Figure S4B,D), a higher NSTS background would have been expected, which is not the case (Figure 9A,C): the level of fluorescence provided by negative control NSTS remains low i.e., is identical to that observed when XD is used instead of HSP (Figure 4, Figure 5, Figure 6, Figure 7 and Figure 8). Two possible and non-mutually exclusive reasons can explain this unexpected result. The first one is related to the larger size of HSP compared to XD, which slows down the diffusion of HSP-CGFP compared to that of XD-CGFP, proportionally decreasing the encounter probability of NGFP with HSP-CGFP, hence leading to a decreased background.

The second reason is tied to the chaperone activity of HSP. NGFP and CGFP being incomplete and hence misfolded proteins, they are natural targets for HSP. By binding misfolded NGFP and CGFP, HSP (be it free or fused to CGFP) would reduce the population of NGFP and CGFP available for binding to each other, which would ultimately result in a lower background. The same reasoning applies to the second negative control NSTH. The reason why NSTH fluorescence level is in the same range as that of the first negative control NSTS is that competitor HSP does not bind HSP-CGFP which, anyway, does not need this binding for being protected against degradation. When NGFP-471 is used in the positive control (N4TS, top left bar plot in Figure 10), NGFP-471 interacts weakly with HSP-CGFP, which produces a fluorescence signal only slightly above NSTS background because of the weak interaction (low affinity) between 471 and HSP. When HSP is added to this weak interaction (N4TH), HSP competes with HSP-CGFP for binding to NGFP-471, which results in a decreased fluorescence with respect to N4TS, but the high NSTS background makes this competition hardly detectable and poorly reliable. Now, when NGFP-hsbMoRE fusion is used instead of NGFP-471 (NMTS, top right bar plot in Figure 10), NGFP-hsbMoRE interacts strongly with HSP-CGFP, which leads to a high fluorescence signal above NSTS and NSTH backgrounds, which remains both low and unchanged as expected. When HSP is added to this strong interaction (NMTH), it competes with HSP-CGFP for binding to NGFP-hsbMoRE, which results in a decreased fluorescence with respect to NMTS.

### 3.5. Competing the hsbMoRE/HSP Interaction Using hsbMoRE as Competitor

**Figure 11:**
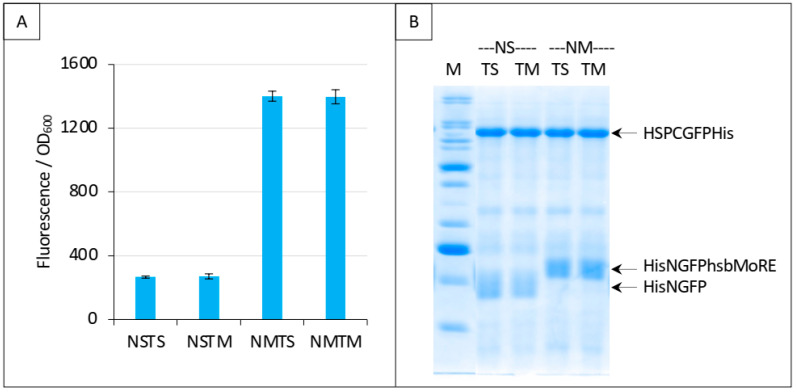
Split-GFP reassembly competition assay using NGFP-hsbMoRE, HSP-CGFP, and hsbMoRE as competitor. Experimental conditions are the same as those described in Figure 9C, except that TH is replaced with TM (hsbMoRE competitor expressed by p17Tet). (**A**) Fluorescence data. (**B**) SDS-PAGE analysis of protein expression. M, molecular mass markers (200, 150, 100, 85, 60, 50, 40, 30, 25, 20, 15, 10 kDa).

Next, we performed the same experiment as that reported in Figure 9C, except that we replaced HSP with hsbMoRE as competitor. Results, reported in Figure 11, show that addition of the competitor did not change the fluorescence. Since we could not detect HishsbMoRE on gel, we cannot exclude that it is not expressed or degraded, which could explain its inability to compete with HisNGFPhsbMoRE for binding XDCGFP (Figure 11A). Using fusion proteins such as thioredoxin-hsbMoRE or using hsbMoRE as a peptide aptamer (i.e., embedded as a loop within a stable protein) [26] might increase the solubility and/or stability of HishsbMoRE, thereby solving this issue.

### 3.6. Assessing the Specificity of the Competition

**Figure 12:**
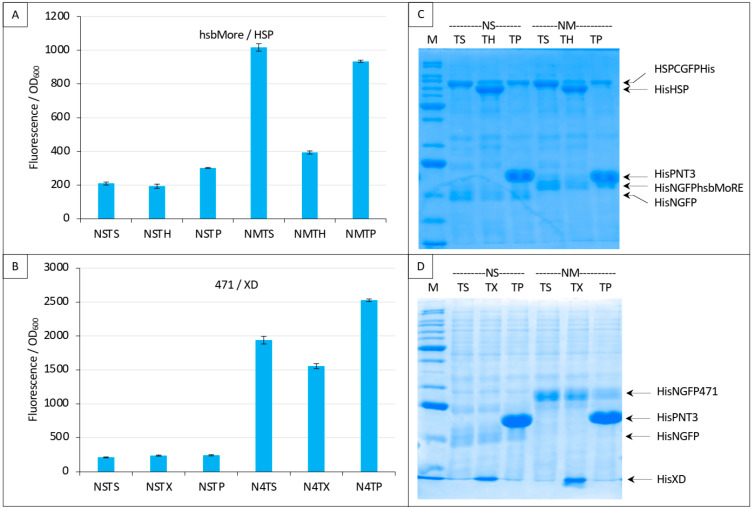
Specificity of the competition using either NGFP-hsbMoRE with HSP-CGFP (**A**,**C**) or NGFP-471 with XD-CGFP (**B**,**D**). (**A**,**B**) Fluorescence data. (**C**,**D**) SDS-PAGE analysis of protein expression. Compared to previous Figures, a third negative control was added with the expression of an irrelevant protein (P, HeV PNT3) as competitor (TP). See main text for details. M, molecular mass markers (200, 150, 100, 85, 60, 50, 40, 30, 25, 20, 15, 10 kDa).

Finally, we evaluated the specificity of the competition obtained with the N_TAIL_/XD pair (Figure 7 and Figure 8) and with the hsbMoRE/HSP pair (Figure 9C and Figure 10). To that end, we used an irrelevant protein as competitor, the PNT3 fragment of the Hendra virus P/V/W protein [24,27]. We also added a new negative control (NSTP) with the competitor (“TP”) co-expressed with NGFP and either CGFP-HSP or CGFP-XD. In line with expectations, results showed that PNT3 was unable to compete with the binding of hsbMoRE to HSP (Figure 12A) or with the binding of 471 to XD (Figure 12B) while it was detectable by SDS-PAGE along with the other proteins of the assay (Figure 12C,D). These results thus provide experimental evidence that the competition we could document using HSP and XD was indeed specific of the interaction involving the two proteins of the split-GFP reassembly assay.

## 4. Discussion

In this work, we aimed at setting up a protein–protein binding competition assay based on the technique of split-GFP reassembly. As a proof of concept, we have used as competitor each of the two interacting proteins of a split-GFP reassembly assay. Results show that a detectable and specific competition can be measured although with some limitations that are discussed below. These results therefore advocate for the suitability of a competition assay based on split-GFP reassembly to compare different variants of one of the two interacting proteins. This would provide an easy way to assess whether one variant has a higher or lower affinity compared to the wild type protein. In addition, since all the proteins are His-tagged, their expression can be checked after fluorescence and biomass data have been recorded.

It should be noted that a non-proteic competitor of the interaction under study could also be used as long as it can cross the *E. coli* cell membrane and is not toxic [28].

A tripartite split-GFP reassembly assay has already been described [28] but, to the best of our knowledge, it has only been used to study positive interactions between three proteins and not for assessing the ability of a third protein to compete for binding with one out of the two interactors, thus conferring originality to our study.

The results reported here show that using a split-GFP reassembly assay for setting up a PPI competition assay is possible but may, under certain circumstances, lead to unexpected, though rationalizable, results. On the basis of the results reported in this study, we conclude that setting up this kind of competition implies several prerequisites: (1) The fluorescence of bacteria co-expressing the three proteins required by the competition assay must be accurately quantified, as we described previously [9]. (2) Both GFP fusions must be stable when expressed alone and must not be protected from degradation by binding to the other interacting partner or to the other GFP half. Should this be the case, a protein unable to protect the degradation-prone protein should be used as competitor. (3) The competitor must be expressed in excess with respect to the GFP-fusion protein it competes with because its binding is reversible whereas the binding of the other GFP half is irreversible, creating a disadvantage for the competitor for long term expression (overnight in the case of the experiments reported in this study). (4) The weakest available GFP variant in terms of both expression level and reassociation affinity must be used (in our case sg100) because it will also provide the best signal to noise ratio for the reasons we have reported previously [9]. In this respect, we have performed with the “folding reporter” (fr) GFP variant the same experiments as those performed with variant sg100 and reported in Figure 4, Figure 5, Figure 6, Figure 7, Figure 8, Figure 9, Figure 10 and Figure 11 (see Supplementary Text S2 and Supplementary Figures S1–S5). In all cases, fr was expressed at higher levels than sg100 and had a higher self-reassociation affinity than sg100 (see Supplementary Figures S1–S5), two features we had already observed [9]. The folding reporter provided, at best, results similar to those obtained with sg100 and, at worse, inconsistent results. For the same reason, protein pairs with low affinity also tend to produce high backgrounds and low signals, which results in unusable or incoherent data. We show that increasing their affinity by mutating one of the two interacting partners improves signal to noise ratio as illustrated by experiments in which 471 was exchanged for hsbMoRE (Figure 9 and Figure 10). Thus, both the affinity of the two GFP halves and that of the two interacting proteins under study are at play: high affinity protein pairs will bind to each other faster than the two GFP halves reassociate. That is the reason why, to obtain high signal to noise ratios, it is important to use GFP variants exhibiting the lowest self-reassociation [9] and protein pairs with the highest affinity. Figure 9 and Figure 10 provide a clear example of this affinity competition issue. By switching from 471 to hsbMoRE, the signal to noise ratio of the binding to HSP increased, which allowed detecting a competition effect of HSP (Figure 9A,C). However, and in further support of our conclusions reported above, this was true only for GFP variant sg100 (Figure 9 and Figure 10) but not for GFP variant fr (Supplementary Figure S4) because the latter has a higher self-reassociation affinity than that of hsbMoRE/HSP, even if hsbMoRE has a higher affinity for HSP than 471.

In all the experiments where a competition was observed (Figure 7, Figure 8, Figure 9, Figure 10 and Figure 12, Supplementary Figures S1 and S3), the latter was always partial. This can be explained by two reasons. First, the level of competitor cannot be controlled and is probably not high enough to completely out compete the binding of the two GFP fusions. This drawback could be in principle overcome by using a promoter stronger than the promoters used by the two split-GFP plasmids. Second, the reassociation of GFP is irreversible contrary to that of the competitor because the latter is not fused to a GFP fragment. Consequently, competition is likely to decrease with time, and so shorter incubations (24 h) should be favored to maximize competition effects.

Considered collectively, the data reported in Figure 7, Figure 8, Figure 9, Figure 10 and Figure 12 provide a good definition of the limits within which the competition assay described in this study and based on the reassembly of split-GFP using sg100 variant should be used. When the affinity of the two proteins under study is too low, which is the case for the 471/HSP interaction (Figure 9A), the specific signal (N4TS) is too close to the background to allow any competition to be considered as specific, even if it eventually happens to be real (Figure 9A). The other extreme is illustrated by the interaction between 471 and XD (Figure 7). In that case, the signal to noise ratio is high enough to consider any fluorescence decrease as ascribable to a real competition. However, in such a scenario, if the protein being used as potential competitor has an affinity for one of the two interacting proteins close (or equal) to that of the interacting pair used in the positive control, then high concentrations of competitor are required for detecting the competition, a criterion effectively met by XD expressed from p17Tet-XD (Figure 7B,D and Figure 12D). An intermediate situation between these two extremes, such as the interaction between hsbMoRE and HSP (Figure 9C, Figure 10 and Figure 12A), would be ideal because this interaction has the advantage of being endowed with an affinity high enough to provide a proportionally high signal to noise ratio while being sufficiently moderate to maximize the chances of identifying an effective competitor (i.e., strong interactions are more difficult to target).

## 5. Conclusions

In conclusion, we herein provide the proof of concept of a protein–protein binding competition assay based on the technique of split-GFP reassembly. This assay, not only appears to be well suited to characterize variants of one of the two interacting proteins in terms of their relative affinity toward the partner protein, but should also provide a convenient tool to identify competitors within libraries. From a practical point of view, when screening libraries of protein variants with the goal of identifying competitors, one should first make sure that the signal to noise ratio is high enough to allow any decrease in fluorescence to be considered as the result of a competition. Once this criterion is met, then any variant leading to a drop in fluorescence can be considered as a *bone fide* competitor. As a caveat, we would like to underscore that the expression level imparts an inherent limitation to this technique, as potent competitors might escape detection if they are poorly expressed. This technical limitation can be however fully overcome when screening chemical libraries, as the concentration of the molecules can be finely controlled.

## Data Availability

The data present in the current study are available from the corresponding authors on reasonable request.

## Supplementary Materials

The following supporting information can be downloaded at: https://www.mdpi.com/article/10.3390/biom13020354/s1. Figure S1: Split-GFP reassembly competition assay using NGFP-471, XD-CGFP, and His-471 as competitor; Figure S2: XDCGFP bound to 471 and/or NGFP is protected from degradation as assessed by fluorescence measurements after one night at 17 °C and SDS-PAGE analysis. Figure S3: Split-GFP reassembly competition assay using NGFP-471, XD-CGFP, and His-XD as competitor; Figure S4: Split-GFP reassembly competition assay using NGFP-471 (A, B) or NGFP-hsbMoRE (C, D) with HSP-CGFP, and HSP as competitor; Figure S5: Split-GFP reassembly competition assay using NGFP-hsbMoRE, HSP-CGFP, and hsbMoRE as competitor; Text S1: features and sequence of the p17Tet vector; Text S2: description of results obtained with fr GFP variant.
