# Supplementary material for: In Vivo Protein–Protein Binding Competition Assay Based on Split-GFP Reassembly: Proof of Concept
Source: Biomolecules. 2023 Feb 11;13(2):354. doi: 10.3390/biom13020354 (PMC9952896; doi:10.3390/biom13020354)
Supplement: Supplementary file 1 [file biomolecules-13-00354-s001.zip › biomolecules-2206954-supplementary.pdf]

In-vivo **protein-protein binding competition assays based on split-GFP reassembly: proof of concept**

Christophe Bignon * and Sonia Longhi *

Lab. Architecture et Fonction des Macromolécules Biologiques (AFMB), UMR 7257, Aix-Marseille University and Centre National de la Recherche Scientifique (CNRS), 163 Avenue de Luminy, Case 932, 13288 Marseille CEDEX 09, France
Correspondence: Christophe.bignon@univ-amu.fr; Sonia.longhi@univ-amu.fr

Supplementary text S1: features and sequence of the p17Tet vector

```
LOCUS       pDEST17O_I_Tet          8556 bp    DNA        linear       30-SEP-2022
DEFINITION  .
ACCESSION
VERSION
SOURCE      .
  ORGANISM  .
COMMENT     >pDEST17O/I-Tet
COMMENT     ApEinfo:methylated:1
FEATURES             Location/Qualifiers
     misc_feature    445..569
                     /locus_tag="attR1"
     misc_feature    618..1337
                     /locus_tag="ChloR"

     misc_feature    1679..1904
                     /locus_tag="ccdB"

     misc_feature    2025..2149
                     /locus_tag="attR2"
                     "
     misc_feature    2633..2638
                     /locus_tag="HindIII"

     misc_feature    2694..4603
                     /locus_tag="TetRTetA_operon"

     misc_feature    7405..8364
                     /locus_tag="LacI"

     misc_feature    418..435
                     /locus_tag="His-tag"
     misc_feature    337..362
                     /locus_tag="LacO"

     misc_feature    318..334
                     /locus_tag="T7prom"
                     /
ORIGIN
        1 GATGGTGTCC GGGATCTCGA CGCTCTCCCT TATGCGACTC CTGCATTAGG AAGCAGCCCA
       61 GTAGTAGGTT GAGGCCGTTG AGCACCGCCG CCGCAAGGAA TGGTGCATGC AAGGAGATGG
      121 CGCCCAACAG TCCCCCGGCC ACGGGGCCTG CCACCATACC CACGCCGAAA CAAGCGCTCA
      181 TGAGCCCGAA GTGGCGAGCC CGATCTTCCC CATCGGTGAT GTCGGCGATA TAGGCGCCAG
      241 CAACCGCACC TGTGGCGCCG GTGATGCCGG CCACGATGCG TCCGGCGTAG AGGATCGAGA
      301 TCTCGATCCC GCGAAATTAA TACGACTCAC TATAGGGGAA TTGTGAGCGG ATAACAATTC
      361 CCCTCTAGAA ATAATTTTGT TTAACTTTAA GAAGGAGATA TACATATGTC GTACTACCAT
      421 CACCATCACC ATCACCTCGA ATCAACAAGT TTGTACAAAA AAGCTGAACG AGAAACGTAA
      481 AATGATATAA ATATCAATAT ATTAAATTAG ATTTTGCATA AAAAACAGAC TACATAATAC
      541 TGTAAAACAC AACATATCCA GTCACTATGG CGGCCGCATT AGGCACCCCA GGCTTTACAC
      601 TTTATGCTTC CGGCTCGTAT AATGTGTGGA TTTTGAGTTA GGATCCGTCG AGATTTTCAG
      661 GAGCTAAGGA AGCTAAAATG GAGAAAAAAA TCACTGGATA TACCACCGTT GATATATCCC
      721 AATGGCATCG TAAAGAACAT TTTGAGGCAT TTCAGTCAGT TGCTCAATGT ACCTATAACC
      781 AGACCGTTCA GCTGGATATT ACGGCCTTTT TAAAGACCGT AAAGAAAAAT AAGCACAAGT
      841 TTTATCCGGC CTTTATTCAC ATTCTTGCCC GCCTGATGAA TGCTCATCCG GAATTCCGTA
      901 TGGCAATGAA AGACGGTGAG CTGGTGATAT GGGATAGTGT TCACCCTTGT TACACCGTTT
      961 TCCATGAGCA AACTGAAACG TTTTCATCGC TCTGGAGTGA ATACCACGAC GATTTCCGGC
     1021 AGTTTCTACA CATATATTCG CAAGATGTGG CGTGTTACGG TGAAAACCTG GCCTATTTCC
     1081 CTAAAGGGTT TATTGAGAAT ATGTTTTTCG TCTCAGCCAA TCCCTGGGTG AGTTTCACCA
```

1141 GTTTTGATTT AAACGTGGCC AATATGGACA ACTTCTTCGC CCCCGTTTTC ACCATGGGCA
1201 AATATTATAC GCAAGGCGAC AAGGTGCTGA TGCCGCTGGC GATTCAGGTT CATCATGCCG
1261 TCTGTGATGG CTTCCATGTC GGCAGAATGC TTAATGAATT ACAACAGTAC TGCGATGAGT
1321 GGCAGGGCGG GGCGTAAAGA TCTGGATCCG GCTTACTAAA AGCCAGATAA CAGTATGCGT
1381 ATTTGCGCGC TGATTTTTGC GGTATAAGAA TATATACTGA TATGTATACC CGAAGTATGT
1441 CAAAAAGAGG TGTGCTATGA AGCAGCGTAT TACAGTGACA GTTGACAGCG ACAGCTATCA
1501 GTTGCTCAAG GCATATATGA TGTCAATATC TCCGGTCTGG TAAGCACAAC CATGCAGAAT
1561 GAAGCCCGTC GTCTGCGTGC CGAACGCTGG AAAGCGGAAA ATCAGGAAGG GATGGCTGAG
1621 GTCGCCCGGT TTATTGAAAT GAACGGCTCT TTTGCTGACG AGAACAGGGA CTGGTGAAAT
1681 GCAGTTTAAG GTTTACACCT ATAAAAGAGA GAGCCGTTAT CGTCTGTTTG TGGATGTACA
1741 GAGTGATATT ATTGACACGC CCGGGCGACG GATGGTGATC CCCCTGGCCA GTGCACGTCT
1801 GCTGTCAGAT AAAGTCTCCC GTGAACTTTA CCCGGTGGTG CATATCGGGG ATGAAAGCTG
1861 GCGCATGATG ACCACCGATA TGGCCAGTGT GCCGGTCTCC GTTATCGGGG AAGAAGTGGC
1921 TGATCTCAGC CACCGCGAAA ATGACATCAA AAACGCCATT AACCTGATGT TCTGGGGAAT
1981 ATAAATGTCA GGCTCCCTTA TACACAGCCA GTCTGCAGGT CGACCATAGT GACTGGATAT
2041 GTTGTGTTTT ACAGTATTAT GTAGTCTGTT TTTTATGCAA AATCTAATTT AATATATTGA
2101 TATTTATATC ATTTTACGTT TCTCGTTCAG CTTTCTTGTA CAAAGTGGTT GATTCGAGGC
2161 TGCTAACAAA GCCCGAAAGG AAGCTGAGTT GGCTGCTGCC ACCGCTGAGC AATAACTAGC
2221 ATAACCCCTT GGGGCCTCTA AACGGGTCTT GAGGGGTTTT TTGCTGAAAG GAGGAACTAT
2281 ATCCGGATAT CCACAGGACG GGTGTGGTCG CCATGATCGC GTAGTCGATA GTGGCTCCAA
2341 GTAGCGAAGC GAGCAGGACT GGGCGGCGGC CAAAGCGGTC GGACAGTGCT CCGAGAACGG
2401 GTGCGCATAG AAATTGCATC AACGCATATA GCGCTAGCAG CACGCCATAG TGACTGGCGA
2461 TGCTGTCGGA ATGGACGATA TCCCGCAAGA GGCCCGGCAG TACCGGCATA ACCAAGCCTA
2521 TGCCTACAGC ATCCAGGGTG ACGGTGCCGA GGATGACGAT GAGCGCATTG TTAGATTTCA
2581 TACACGGTGC CTGACTGCGT TAGCAATTTA ACTGTGATAA ACTACCGCAT TAAAGCTTCT
2641 CGACATCTTG GTTACCGTGA AGTTACCATC ACGGAAAAAG GTTATGCTGC TTTTAAGACC
2701 CACTTTCACA TTTAAGTTGT TTTTCTAATC CGCATATGAT CAATTCAAGG CCGAATAAGA
2761 AGGCTGGCTC TGCACCTTGG TGATCAAATA ATTCGATAGC TTGTCGTAAT AATGGCGGCA
2821 TACTATCAGT AGTAGGTGTT TCCCTTTCTT CTTTAGCGAC TTGATGCTCT TGATCTTCCA
2881 ATACGCAACC TAAAGTAAAA TGCCCCACAG CGCTGAGTGC ATATAATGCA TTCTCTAGTG
2941 AAAAACCTTG TTGGCATAAA AAGGCTAATT GATTTTCGAG AGTTTCATAC TGTTTTTCTG
3001 TAGGCCGTGT ACCTAAATGT ACTTTTGCTC CATCGCGATG ACTTAGTAAA GCACATCTAA
3061 AACTTTTAGC GTTATTACGT AAAAAATCTT GCCAGCTTTC CCCTTCTAAA GGGCAAAAGT
3121 GAGTATGGTG CCTATCTAAC ATCTCAATGG CTAAGGCGTC GAGCAAAGCC CGCTTATTTT
3181 TTACATGCCA ATACAATGTA GGCTGCTCTA CACCTAGCTT CTGGGCGAGT TTACGGGTTG
3241 TTAAACCTTC GATTCCGACC TCATTAAGCA GCTCTAATGC GCTGTTAATC ACTTTACTTT
3301 TATCTAATCT AGACATCATT AATTCCTAAT TTTTGTTGAC ACTCTATCAT TGATAGAGTT
3361 ATTTTACCAC TCCCTATCAG TGATAGAGAA AAGTGAAATG AATAGTTCGA CAAAGATCGC
3421 ATTGGTAATT ACGTTACTCG ATGCCATGGG GATTGGCCTT ATCATGCCAG TCTTGCCAAC
3481 GTTATTACGT GAATTTATTG CTTCGGAAGA TATCGCTAAC CACTTTGGCG TATTGCTTGC
3541 ACTTTATGCG TTAATGCAGG TTATCTTTGC TCCTTGGCTT GGAAAAATGT CTGACCGATT
3601 TGGTCGGCGC CCAGTGCTGT TGTTGTCATT AATAGGCGCA TCGCTGGATT ACTTATTGCT
3661 GGCTTTTTCA AGTGCGCTTT GGATGCTGTA TTTAGGCCGT TTGCTTTCAG GGATCACAGG
3721 AGCTACTGGG GCTGTCGCGG CATCGGTCAT TGCCGATACC ACCTCAGCTT CTCAACGCGT
3781 GAAGTGGTTC GGTTGGTTAG GGGCAAGTTT TGGGCTTGGT TTAATAGCGG GGCCTATTAT
3841 TGGTGGTTTT GCAGGAGAGA TTTCACCGCA TAGTCCCTTT TTTATCGCTG CGTTGCTAAA
3901 TATTGTCACT TTCCTTGTGG TTATGTTTTG GTTCCGTGAA ACCAAAAATA CACGTGATAA
3961 TACAGATACC GAAGTAGGGG TTGAGACGCA ATCGAATTCG GTATACATCA CTTTATTTAA
4021 AACGATGCCC ATTTTGTTGA TTATTTATTT TTCAGCGCAA TTGATAGGCC AAATTCCCGC
4081 AACGGTGTGG GTGCTATTTA CCGAAAATCG TTTTGGATGG AATAGCATGA TGGTTGGCTT
4141 TTCATTAGCG GGTCTTGGTC TTTTACACTC AGTATTCCAA GCCTTTGTGG CAGGAAGAAT
4201 AGCCACTAAA TGGGGCGAAA AAACGGCAGT ACTGCTCGAA TTTATTGCAG ATAGTAGTGC
4261 ATTTGCCTTT TTAGCGTTTA TATCTGAAGG TTGGTTAGAT TTCCCTGTTT TAATTTTATT
4321 GGCTGGTGGT GGGATCGCTT TACCTGCATT ACAGGGAGTG ATGTCTATCC AAACAAAGAG
4381 TCATGAGCAA GGTGCTTTAC AGGGATTATT GGTGAGCCTT ACCAATGCAA CCGGTGTTAT
4441 TGGCCCATTA CTGTTTACTG TTATTTATAA TCATTCACTA CCAATTTGGG ATGGCTGGAT
4501 TTGGATTATT GGTTTAGCGT TTTACTGTAT TATTATCCTG CTATCGATGA CCTTCATGTT
4561 AACCCCTCAA GCTCAGGGGA GTAAACAGGA GACAAGTGCT TAGTTATTTC GTCACCAAAT
4621 GATGTTATTC CGCGGACGGG GAGTCAGGCA ACTATGGATG AACGAAATAG ACAGATCGCT
4681 GAGATAGGTG CCTCACTGAT TAAGCATTGG TAACTGTCAG ACCAAGTTTA CTCATATATA
4741 CTTTAGATTG ATTTAAAACT TCATTTTTAA TTTAAAAGGA TCTAGGTGAA GATCCTTTTT
4801 GATAATCTCA TGACCAAAAT CCCTTAACGT GAGTTTTCGT TCCACTGAGC GTCAGACCCC
4861 GTAGAAAAGA TCAAAGGATC TTCTTGAGAT CCTTTTTTTC TGCGCGTAAT CTGCTGCTTG
4921 CAAACAAAAA AACCACCGCT ACCAGCGGTG GTTTGTTTGC CGGATCAAGA GCTACCAACT
4981 CTTTTTCCGA AGGTAACTGG CTTCAGCAGA GCGCAGATAC CAAATACTGT CCTTCTAGTG
5041 TAGCCGTAGT TAGGCCACCA CTTCAAGAAC TCTGTAGCAC CGCCTACATA CCTCGCTCTG
5101 CTAATCCTGT TACCAGTGGC TGCTGCCAGT GGCGATAAGT CGTGTCTTAC CGGGTTGGAC
5161 TCAAGACGAT AGTTACCGGA TAAGGCGCAG CGGTCGGGCT GAACGGGGGG TTCGTGCACA
5221 CAGCCCAGCT TGGAGCGAAC GACCTACACC GAACTGAGAT ACCTACAGCG TGAGCTATGA
5281 GAAAGCGCCA CGCTTCCCGA AGGGAGAAAG GCGGACAGGT ATCCGGTAAG CGGCAGGGTC
5341 GGAACAGGAG AGCGCACGAG GGAGCTTCCA GGGGGAAACG CCTGGTATCT TTATAGTCCT
5401 GTCGGGTTTC GCCACCTCTG ACTTGAGCGT CGATTTTTGT GATGCTCGTC AGGGGGGCGG
5461 AGCCTATGGA AAAACGCCAG CAACGCGGCC TTTTTACGGT TCCTGGCCTT TTGCTGGCCT
5521 TTTGCTCACA TGTTCTTTCC TGCGTTATCC CCTGATTCTG TGGATAACCG TATTACCGCC
5581 TTTGAGTGAG CTGATACCGC TCGCCGCAGC CGAACGACCG AGCGCAGCGA GTCAGTGAGC
5641 GAGGAAGCGG AAGAGCGCCT GATGCGGTAT TTTCTCCTTA CGCATCTGTG CGGTATTTCA
5701 CACCGCATAT ATGGTGCACT CTCAGTACAA TCTGCTCTGA TGCCGCATAG TTAAGCCAGT

```
5761 ATACACTCCG CTATCGCTAC GTGACTGGGT CATGGCTGCG CCCCGACACC CGCCAACACC
5821 CGCTGACGCG CCCTGACGGG CTTGTCTGCT CCCGGCATCC GCTTACAGAC AAGCTGTGAC
5881 CGTCTCCGGG AGCTGCATGT GTCAGAGGTT TTCACCGTCA TCACCGAAAC GCGCGAGGCA
5941 GCTGCGGTAA AGCTCATCAG CGTGGTCGTG AAGCGATTCA CAGATGTCTG CCTGTTCATC
6001 CGCGTCCAGC TCGTTGAGTT TCTCCAGAAG CGTTAATGTC TGGCTTCTGA TAAAGCGGGC
6061 CATGTTAAGG GCGGTTTTTT CCTGTTTGGT CACTGATGCC TCCGTGTAAG GGGGATTTCT
6121 GTTCATGGGG GTAATGATAC CGATGAAACG AGAGAGGATG CTCACGATAC GGGTTACTGA
6181 TGATGAACAT GCCCGGTTAC TGGAACGTTG TGAGGGTAAA CAACTGGCGG TATGGATGCG
6241 GCGGGACCAG AGAAAAATCA CTCAGGGTCA ATGCCAGCGC TTCGTTAATA CAGATGTAGG
6301 TGTTCCACAG GGTAGCCAGC AGCATCCTGC GATGCAGATC CGGAACATAA TGGTGCAGGG
6361 CGCTGACTTC CGCGTTTCCA GACTTTACGA AACACGGAAA CCGAAGACCA TTCATGTTGT
6421 TGCTCAGGTC GCAGACGTTT TGCAGCAGCA GTCGCTTCAC GTTCGCTCGC GTATCGGTGA
6481 TTCATTCTGC TAACCAGTAA GGCAACCCCG CCAGCCTAGC CGGGTCCTCA ACGACAGGAG
6541 CACGATCATG CGCACCCGTG GCCAGGACCC AACGCTGCCC GAGATGCGCC GCGTGCGGCT
6601 GCTGGAGATG GCGGACGCGA TGGATATGTT CTGCCAAGGG TTGGTTTGCG CATTCACAGT
6661 TCTCCGCAAG AATTGATTGG CTCCAATTCT TGGAGTGGTG AATCCGTTAG CGAGGTGCCG
6721 CCGGCTTCCA TTCAGGTCGA GGTGGCCCGG CTCCATGCAC CGCGACGCAA CGCGGGGAGG
6781 CAGACAAGGT ATAGGGCGGC GCCTACAATC CATGCCAACC CGTTCCATGT GCTCGCCGAG
6841 GCGGCATAAA TCGCCGTGAC GATCAGCGGT CCAGTGATCG AAGTTAGGCT GGTAAGAGCC
6901 GCGAGCGATC CTTGAAGCTG TCCCTGATGG TCGTCATCTA CCTGCCTGGA CAGCATGGCC
6961 TGCAACGCGG GCATCCCGAT GCCGCCGGAA GCGAGAAGAA TCATAATGGG GAAGGCCATC
7021 CAGCCTCGCG TCGCGAACGC CAGCAAGACG TAGCCCAGCG CGTCGGCCGC CATGCCGGCG
7081 ATAATGGCCT GCTTCTCGCC GAAACGTTTG GTGGCGGGAC CAGTGACGAA GGCTTGAGCG
7141 AGGGCGTGCA AGATTCCGAA TACCGCAAGC GACAGGCCGA TCATCGTCGC GCTCCAGCGA
7201 AAGCGGTCCT CGCCGAAAAT GACCCAGAGC GCTGCCGGCA CCTGTCCTAC GAGTTGCATG
7261 ATAAAGAAGA CAGTCATAAG TGCGGCGACG ATAGTCATGC CCCGCGCCCA CCGGAAGGAG
7321 CTGACTGGGT TGAAGGCTCT CAAGGGCATC GGTCGAGATC CCGGTGCCTA ATGAGTGAGC
7381 TAACTTACAT TAATTGCGTT GCGCTCACTG CCCGCTTTCC AGTCGGGAAA CCTGTCGTGC
7441 CAGCTGCATT AATGAATCGG CCAACGCGCG GGGAGAGGCG GTTTGCGTAT TGGGCGCCAG
7501 GGTGGTTTTT CTTTTCACCA GTGAGACGGG CAACAGCTGA TTGCCCTTCA CCGCCTGGCC
7561 CTGAGAGAGT TGCAGCAAGC GGTCCACGCT GGTTTGCCCC AGCAGGCGAA AATCCTGTTT
7621 GATGGTGGTT AACGGCGGGA TATAACATGA GCTGTCTTCG GTATCGTCGT ATCCCACTAC
7681 CGAGATATCC GCACCAACGC GCAGCCCGGA CTCGGTAATG GCGCGCATTG CGCCCAGCGC
7741 CATCTGATCG TTGGCAACCA GCATCGCAGT GGGAACGATG CCCTCATTCA GCATTTGCAT
7801 GGTTTGTTGA AAACCGGACA TGGCACTCCA GTCGCCTTCC CGTTCCGCTA TCGGCTGAAT
7861 TTGATTGCGA GTGAGATATT TATGCCAGCC AGCCAGACGC AGACGCGCCG AGACAGAACT
7921 TAATGGGCCC GCTAACAGCG CGATTTGCTG GTGACCCAAT GCGACCAGAT GCTCCACGCC
7981 CAGTCGCGTA CCGTCTTCAT GGGAGAAAAT AATACTGTTG ATGGGTGTCT GGTCAGAGAC
8041 ATCAAGAAAT AACGCCGGAA CATTAGTGCA GGCAGCTTCC ACAGCAATGG CATCCTGGTC
8101 ATCCAGCGGA TAGTTAATGA TCAGCCCACT GACGCGTTGC GCGAGAAGAT TGTGCACCGC
8161 CGCTTTACAG GCTTCGACGC CGCTTCGTTC TACCATCGAC ACCACCACGC TGGCACCCAG
8221 TTGATCGGCG CGAGATTTAA TCGCCGCGAC AATTTGCGAC GGCGCGTGCA GGGCCAGACT
8281 GGAGGTGGCA ACGCCAATCA GCAACGACTG TTTGCCCGCC AGTTGTTGTG CCACGCGGTT
8341 GGGAATGTAA TTCAGCTCCG CCATCGCCGC TTCCACTTTT TCCCGCGTTT TCGCAGAAAC
8401 GTGGCTGGCC TGGTTCACCA CGCGGGAAAC GGTCTGATAA GAGACACCGG CATACTCTGC
8461 GACATCGTAT AACGTTACTG GTTTCACATT CACCACCCTG AATTGACTCT CTTCCGGGCG
8521 CTATCATGCC ATACCGCGAA AGGTTTTGCG CCATTC
//
```

Supplementary text S2: description of results obtained with fr GFP variant

All the experiments using fr (folding reporter [1]) have been run in parallel with those using sg100 and so experimental conditions described for sg100 also apply to fr.

Results reported in **Supplementary Figure S1**~~,~~ correspond to those of sg100 in **Figure 2**. As for sg100, fr provided the same two types of results but overall, the data were less reproducible and essentially non-coherent (**Supplementary Figure S1A** and **B**). The only interest of these results was that XD-CfrGFP was his-tagged contrary to XD-Csg100GFP as used in **Figure 2**, which allowed its detection on gels (**Supplementary Figure S1C** and **D**) and the observation of a proportionality between fluorescence data and XD-CfrGFP band intensity on gel.

In the same vein as Figure 3 for sg100, **Supplementary Figure S2** provides an explanation of the data in **Supplementary Figure S1**, which leads to the same conclusion.

For fr, **Supplementary Figure S3** is the equivalent of Figure 5 for sg100: XD was used as competitor instead of 471 with similar conclusions. However, while competition was observed in all cases when sg100 was used, it was so only in some cases for fr, suggesting that fr is less reliable than sg100 for setting up a competition assay based on split-GFP reassembly.

Supplementary Figure S4 is the equivalent for fr of **Figure 7** for sg100. However, as one can see, it provided incoherent results.

The same can be said for **Supplementary Figure S5**, which is the equivalent of **Figure 9** for sg100 and provided incoherent results. An explanation for the incoherent nature of the results obtained with fr, can be found in main text (discussion section).

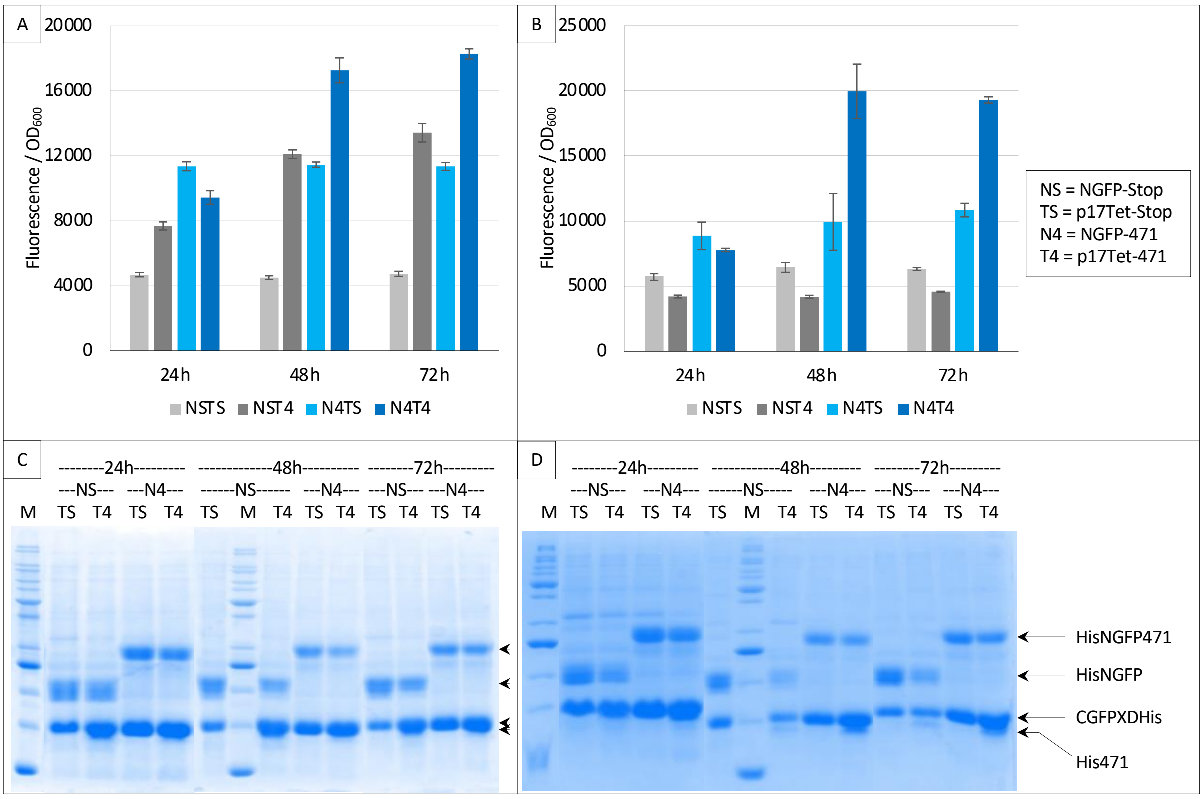

Figure S1 Split-GFP reassembly competition assay using NGFP-471, XD-CGFP, and His-471 as competitor. A and B are two independent experiments, each performed in triplicate for 24, 48 or 72h. NSTS and NST4 are the two negative controls. See main text for a description of NSTS, NST4, N4TS and N4T4. C and D are SDS-PAGE of A and B, respectively. M, molecular mass markers (200, 150, 100, 85, 60, **50**, 40, 30, **25**, 20, 15, 10 kDa).

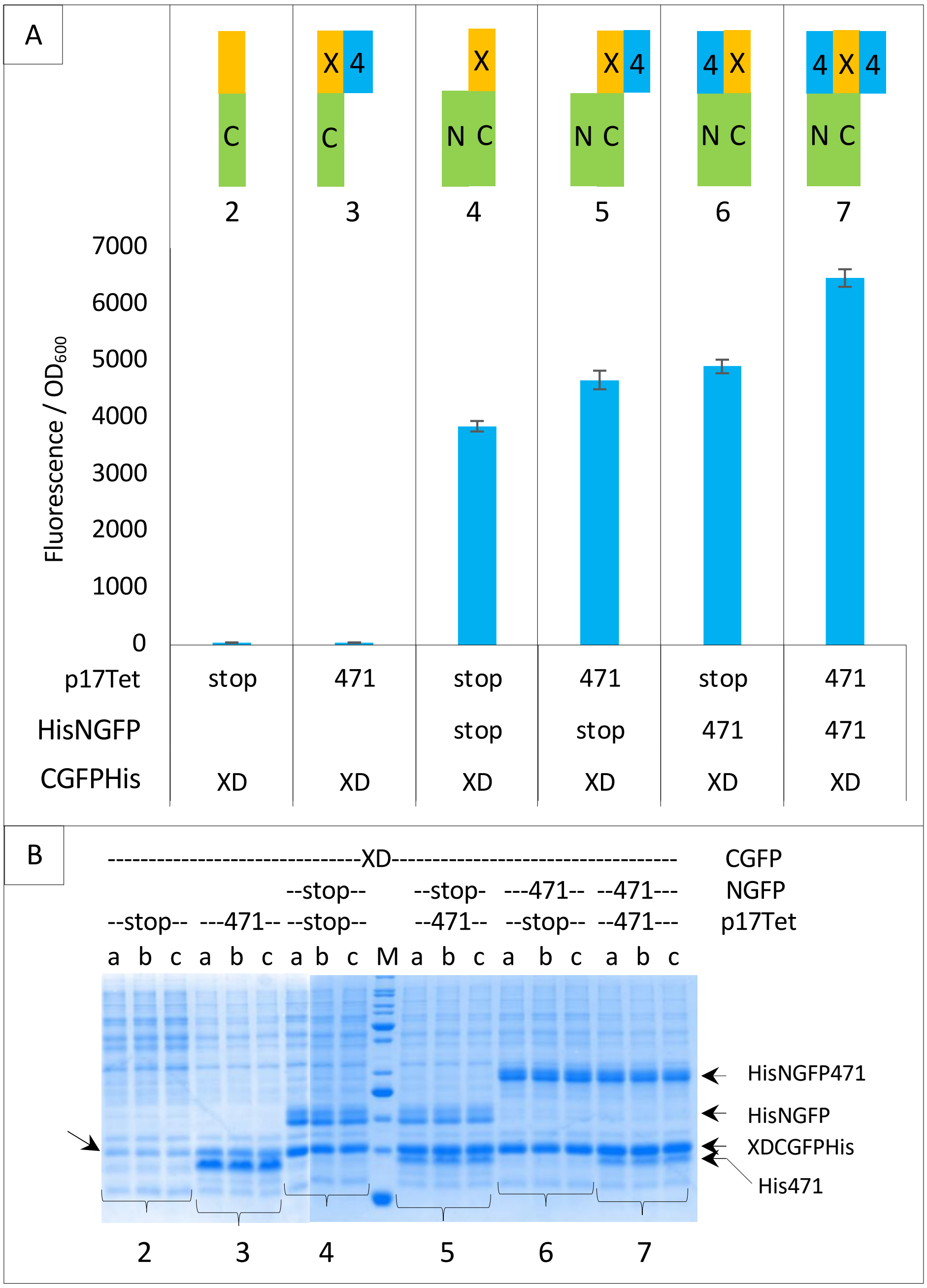

Figure S2. XDCGFP bound to 471 and / or NGFP is protected from degradation as assessed by fluorescence measurements after one night at 17°C and SDS-PAGE analysis. A, top. The different proteins and protein combinations, numbered from 2 to 7, are illustrated by colored rectangles: 471 = 4 in blue rectangle, XD = X in yellow rectangle, CGFP = C in green rectangle, NGFP = N in green rectangle. Fusion proteins XC and 4N are represented as fused rectangles. To avoid ambiguity, competitor 471 bound to XD is arbitrarily represented on the right of XD, whereas NGFP471 bound to XDCGFP is arbitrarily represented on the left of XDCGFP. A, bottom. The fluorescence data obtained with conditions 2 to 7 are shown below the corresponding protein or protein combinations. B, SDS-PAGE analysis of proteins expressed in A. Triplicates were loaded individually (a, b, c) to assess the reproducibility of each loading. The lowest steady-state level of XD-CGFP is indicated by an oblique arrow (condition 2). Numbers 2 to 7 below the gels refer to the different combinations of panel A. The different fusion proteins are indicated on the right of the gels. M, molecular mass markers (200, 150, 100, 85, 60, **50**, 40, 30, **25**, 20, 15, 10 kDa).

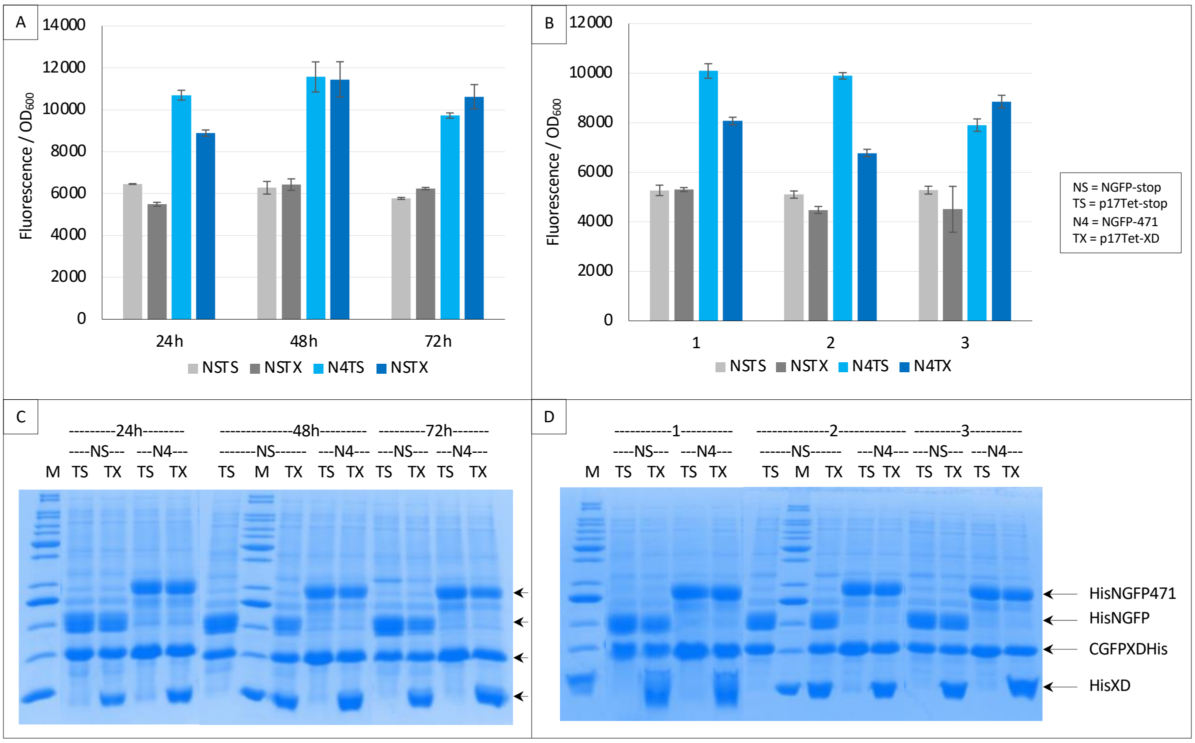

Figure S3 Split-GFP reassembly competition assay using NGFP-471, XD-CGFP, and His-XD as competitor. A, experiment performed in triplicate for 24, 48 or 72h. NSTS and NSTX are the two negative controls. TX is free XD used as competitor. See main text for a description of NSTS, NSTX, N4TS and N4TX. B, three independent experiments (labeled 1, 2, 3 on the X-axis), each performed in triplicate and for 24h. C and D are SDS-PAGE analysis of A and B, respectively. M, molecular mass markers (200, 150, 100, 85, 60, **50**, 40, 30, **25**, 20, 15, 10 kDa).

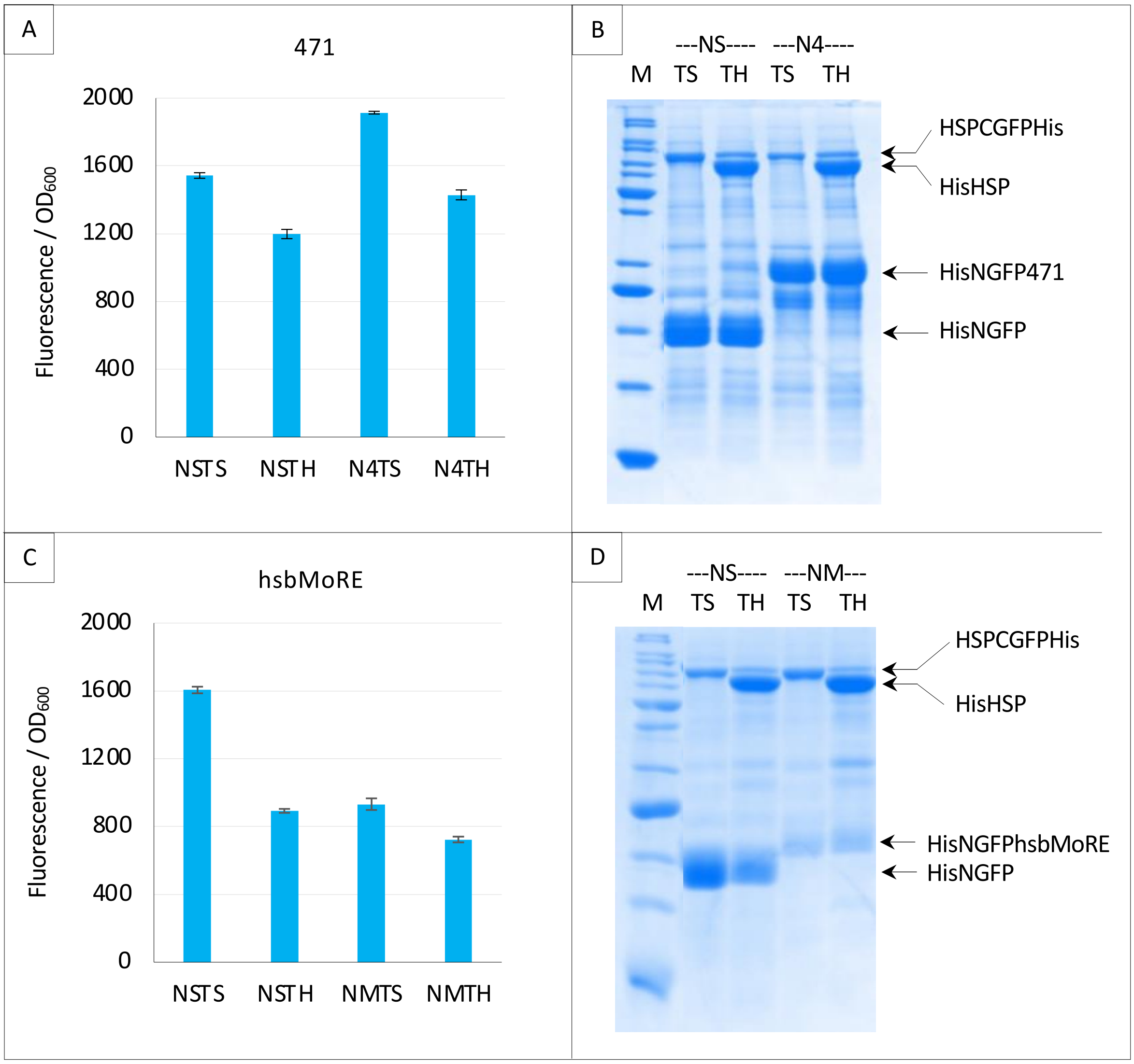

Figure S4. Split-GFP reassembly competition assay using NGFP-471 (A, B) or NGFP-hsbMoRE (C, D) with HSP-CGFP, and HSP as competitor. A and C. Fluorescence data. B and D are SDS-PAGE analysis of protein expression. See main text for a description of NSTS, NSTH, N4TS and N4TH. M, molecular mass markers (200, 150, 100, 85, 60, **50**, 40, 30, **25**, 20, 15, 10 kDa).

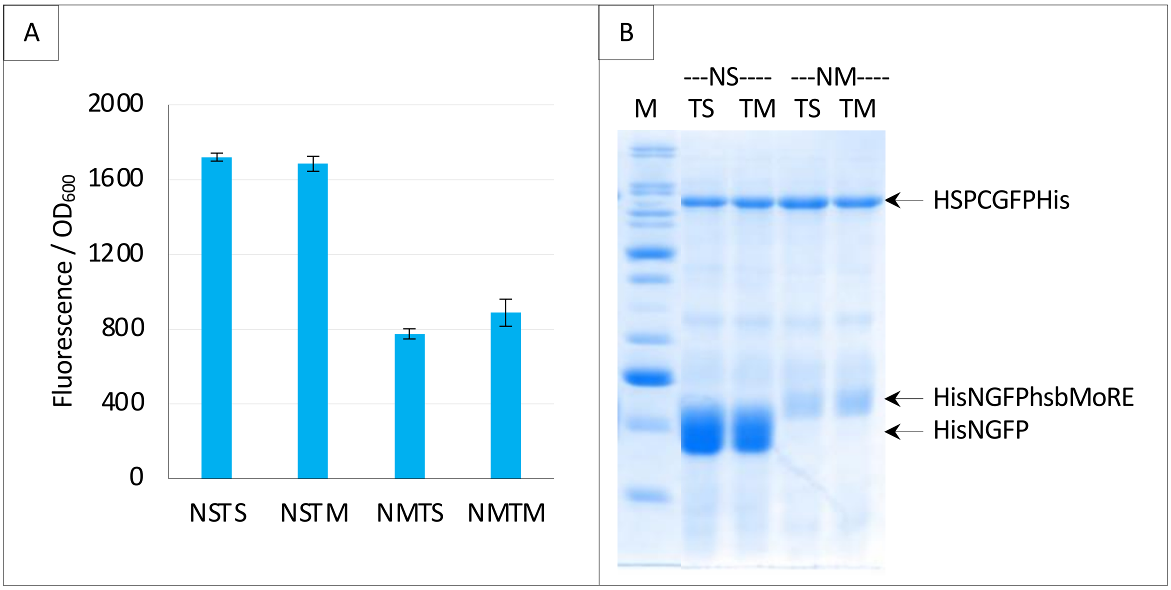

Figure S5. Split-GFP reassembly competition assay using NGFP-hsbMoRE, HSP-CGFP, and hsbMoRE as competitor. Experimental conditions are the same as those described in Figure S4, except that TH is replaced with TM (hsbMoRE competitor expressed by p17Tet). A, Fluorescence data. B, SDS-PAGE analysis of protein expression. M, molecular mass markers (200, 150, 100, 85, 60, 50, 40, 30, 25, 20, 15, 10 kDa).

References

1. Waldo, G.S.; Standish, B.M.; Berendzen, J.; Terwilliger, T.C. Rapid protein-folding assay using green fluorescent protein. *Nat. Biotechnol.* **1999**, *17*, 691-695, doi:10.1038/10904.
